# The spatiotemporal evolution mechanism of enterprise-led organic agriculture: Evidence from Shandong Province

**DOI:** 10.1371/journal.pone.0362492

**Published:** 2026-11-11

**Authors:** Yueling Yang, Qiang Wang, Shu Wang, Zihong Yang, Thomas Dogot

**Affiliations:** 1 Department of Economics and Rural Development, Gembloux Agro-Bio Tech, University of Liège, Gembloux, Belgium; 2 School of Business, Shandong Management University, Jinanq, China; 3 Jinan University—University of Birmingham Joint Institute, Jinan University, Guangzhou, China; 4 Institute of Agricultural Resources and Regional Planning, Chinese Academy of Agricultural Sciences, Beijing, China; Dongshin University, KOREA, REPUBLIC OF

## Abstract

This study investigates the spatiotemporal evolution and driving mechanisms of enterprise-led organic agriculture in Shandong Province, China. Using panel data on certified organic enterprise production bases across 16 prefecture-level cities from 2014 to 2023, we combine spatial statistical analysis, Local Moran’s I, two-way fixed-effects regression, and robustness checks. The results show that certified organic bases expanded substantially but became increasingly concentrated, forming a clear core–periphery structure centered on the Jiaodong Peninsula. Transportation infrastructure, e-commerce connectivity, and GI-protected agricultural products are positively associated with base expansion, while fiscal expenditure shows a negative association, possibly reflecting compensatory policy allocation to less-developed areas. Overall, the findings suggest that enterprise-led organic agriculture evolves through a layered “Pressure–Transformation–Support–Market” mechanism and provide evidence for promoting coordinated regional development in sustainable agriculture.

## 1 Introduction

With the rising global momentum of sustainable development and the growing demand for food safety, organic agriculture has become a strategic pathway for the transformation and upgrading of modern agriculture. First, organic agriculture makes outstanding contributions to ecological protection, the sustainable use of resources, and food safety. Organic agriculture is “a holistic production management system that promotes ecosystem health, biodiversity, and soil biological activity” [1]. Second, the Chinese government is promoting organic agriculture with unprecedented policy efforts. As a traditional agricultural powerhouse, China is vigorously advancing the transformation and upgrading of its conventional agriculture. The area of organic farmland has increased from 1.16 million hectares in 2013 to more than 4 million hectares in 2023, ranking fourth worldwide [2]. Third, the continuously expanding market highlights the enormous development potential and strategic significance of organic agriculture. In 2023, the global retail sales of organic agricultural products approached €136 billion, a year-on-year increase of 1.6%. Among them, China, with a market size of €12.6 billion, ranked as the world's third largest organic market for the second consecutive year [3].

Organic agriculture in China is primarily driven by enterprises. Organic conversion involves long certification periods, high upfront costs, stringent standards, and substantial market uncertainty [4]. Smallholders often lack the financial resources and managerial capacity to assume such risks. Consequently, enterprises have become the core forces driving the expansion of organic production through investment in production bases located in regions with favorable industrial, market, and policy conditions. This firm-driven model leads to an “organization-first, land-later” transition path in China’s organic agriculture, resulting in pronounced spatial selectivity and regional clustering [5]. Therefore, this study focuses on certified organic production bases rather than organic farmland area, as the former serve as the organizational and market entities that substantially shape the spatial expansion of the organic sector in China.

Shandong Province, as both an agricultural giant and a core vegetable-producing region in China, holds national-level demonstration value for agricultural transformation. First, Shandong’s strategic position in Chinese agriculture is notable. It possesses extensive cultivated land, and its agricultural output value has long ranked among the top nationwide, playing a crucial role in ensuring national food security and agricultural supply [6]. Second, Shandong Province is prioritizing the development of organic agriculture as a key lever for achieving high-quality agricultural development and ecological revitalization of rural areas. With the introduction of numerous supportive policies, the number of certified organic enterprises and products in Shandong has grown significantly. A number of well-known organic production bases have been established. Organic production bases refer to production, planting, or breeding bases owned by enterprises and officially certified [7]. Organic production bases covering diverse categories and forming an initial pattern of regionalized and specialized development. Third, the rapid increase in the number of certified bases has masked the widening spatial disparities between regions in Shandong Province. These disparities may lead to uneven distribution of policy support, reduced market efficiency, and an unbalanced transition to sustainable agriculture [8].. Beneath the surface of rapid expansion, such heterogeneity is profoundly influencing the overall effectiveness and sustainability of development. Therefore, merely describing spatial distribution is insufficient to understand the true state of organic agriculture development in Shandong Province. More crucially, it is essential to reveal the underlying enterprise-driven spatiotemporal evolution mechanism and the intrinsic driving force behind the increasing regional imbalances, in order to support more precise industrial policy formulation. This is of great significance for enhancing the resilience and synergy of the organic agriculture system.

The development of organic agriculture can be conceptualized from two complementary theoretical perspectives. First, environmental regulation and industrial transformation theories. In particular, the Porter Hypothesis [9] suggests that the high-intensity use of agrochemicals in conventional agriculture generates substantial environmental stress, which triggers regulatory and market-driven demands for green transition [10]. In this context, enterprises are incentivized to upgrade toward organic production through certification systems and policy support. Second, spatial economics and agglomeration theories. They highlight that improved accessibility—through transportation networks, digital infrastructure, and market capacity—reduces transaction costs and enhances market potential [11]. Advantageous regions therefore attract more investment, benefiting from scale economies, knowledge spillovers, and branding effects [12]. This leads to persistent core–periphery spatial structures within a province. Based on these theoretical foundations, we argue that the spatial layout of certified organic production bases is shaped by institutional responses to environmental pressures and further strengthened by market accessibility and agglomeration externalities, resulting in continuously increasing spatial heterogeneity [13].

A substantial body of research has accumulated on organic agriculture at both international and domestic levels. Existing studies have primarily examined two aspects: spatial distribution and driving factors. On the one hand, many scholars have explored the spatial patterns of organic farming and their evolution at national, regional, or local scales [14]. For example, European and North American studies often highlight clustering effects in areas with strong environmental regulation [15], consumer demand [16], and market accessibility [17], whereas developing countries tend to emphasize regional disparities and resource constraints [18,19]. On the other hand, research on influencing factors has adopted highly diverse sets of variables, including natural endowments [20], socioeconomic foundations [21], institutional arrangements [22], and market-related drivers [23]. However, there is no unified standard for indicator selection, and the types of factors considered vary widely across studies, leading to fragmented conclusions.

Previous studies have established that agricultural and environmental development in Shandong Province exhibits pronounced spatial and temporal heterogeneity. Liu et al. (2020) examined county-level agricultural multifunctionality and showed that production, economic, social-security, and ecological functions followed different spatial trajectories under heterogeneous resource endowments and socioeconomic conditions [24]. Xu et al. (2020) evaluated agricultural eco-efficiency across 134 counties and examined its temporal-spatial differences and influencing factors. From a land-use perspective [25], Ou and Wang (2022) traced the long-term expansion of greenhouse-led cultivated land and demonstrated that its spatial dynamics were associated with rural expenditure, local markets, farmer income, and external vegetable supply and demand [26]. More recent studies have extended this spatial perspective to ecosystem service value and terrestrial carbon source–sink dynamics in Shandong Province [27,28].

Collectively, these studies demonstrate that the spatiotemporal evolution of agriculture and related environmental processes in Shandong has already received substantial scholarly attention. However, their analytical objects primarily concern agricultural functions, eco-efficiency, physical land-use change, or ecological outcomes. These perspectives do not directly capture the spatial organization of formally certified organic production from an enterprise-based perspective. Certified enterprise production bases represent neither organic cultivated area nor production volume; rather, their number and distribution provide an indicator of the organizational presence and formal participation of enterprises within the organic certification system.

Accordingly, the research gap addressed in this study is not a general absence of spatiotemporal research on agricultural development in Shandong, but the limited evidence on how certified enterprise-led organic production bases have evolved and become spatially differentiated across cities over time. This study therefore examines the 16 prefecture-level cities of Shandong from 2014 to 2023 to characterize the temporal evolution and spatial agglomeration of certified enterprise production bases and to assess the regional agricultural, institutional, infrastructural, and market conditions associated with their development. By combining Global and Local Moran’s I with city–year panel analysis, the study provides a certification-based organizational perspective that complements existing land-use, efficiency, functional, and ecological perspectives on regional agricultural transformation.

The contribution of this study is primarily empirical and analytical rather than a claim that spatiotemporal agricultural research in Shandong is itself novel. First, it shifts the analytical object from agricultural land, multifunctionality, eco-efficiency, or ecological outcomes to certified enterprise production bases, thereby providing complementary evidence on the organizational geography of formal organic production. Second, the study distinguishes overall spatial dependence from local clustering by combining Global Moran’s I with LISA analysis, allowing the emergence and persistence of local high–high clusters to be identified. Third, the city–year panel analysis complements the descriptive spatial evidence by examining regional conditions associated with changes in certified-base development after accounting for time-invariant city characteristics and common year shocks. These contributions provide a more specific understanding of the spatial organization of enterprise-led organic agriculture while remaining complementary to, rather than substituting for, existing studies of agricultural and environmental transformation in Shandong Province.

## 2. Materials and methods

### 2.1 Technical framework

This study constructs a three-stage analytical framework composed of “spatiotemporal evolution analysis – spatial correlation test – influencing factor identification.” Multiple methods are integrated, including GIS-based visualization, spatial statistical models, and correlation tests, to form a comprehensive empirical research path.

For visualization, values were classified using the Jenks natural breaks method [29]. Moran’s I was applied to test spatial dependence [30]. Pearson correlation [31] and two-way fixed-effects (TWFE) panel regressions [32] were then used in combination to screen potential determinants and assess robustness.

### 2.2 Data sources

(1) Data on organic agricultural enterprises

The certification records used to construct the dependent variable were obtained from the Qiyan Social Science Big Data Platform, specifically from the Agricultural Research (CCAD) database under “Agricultural Product Research → China Food Safety Research Database → Food and Agricultural Product Certification → Organic Product Certificate Information Table.” The records were extracted for Shandong Province and subsequently cleaned, deduplicated, geocoded to the prefecture-level city, and aggregated by city and year to construct the analytical panel for 2014–2023.

According to regulations, each product certification is valid for one year [33]. Certified organizations should submit a recertification application to the certification body at least three months before the expiry date of the certification certificate. If the certified organization's organic product management system and production/processing procedures remain unchanged, the certification body may appropriately simplify the application review and document review procedures. The total number of certified organic products in China showed a continuous growth trend during 2014–2023. As of March 2024, there were 18,150 certified organic enterprises nationwide, including 31 foreign trade enterprises whose production bases are located overseas. The number of certified product categories reached 46,443, of which 385 were overseas products. In total, there were 24,793 certified enterprise production and processing bases nationwide, with their spatial distribution shown in Fig 1. The city-level dataset and associated explanatory variables are provided in S1 Table.

**Fig 1:**
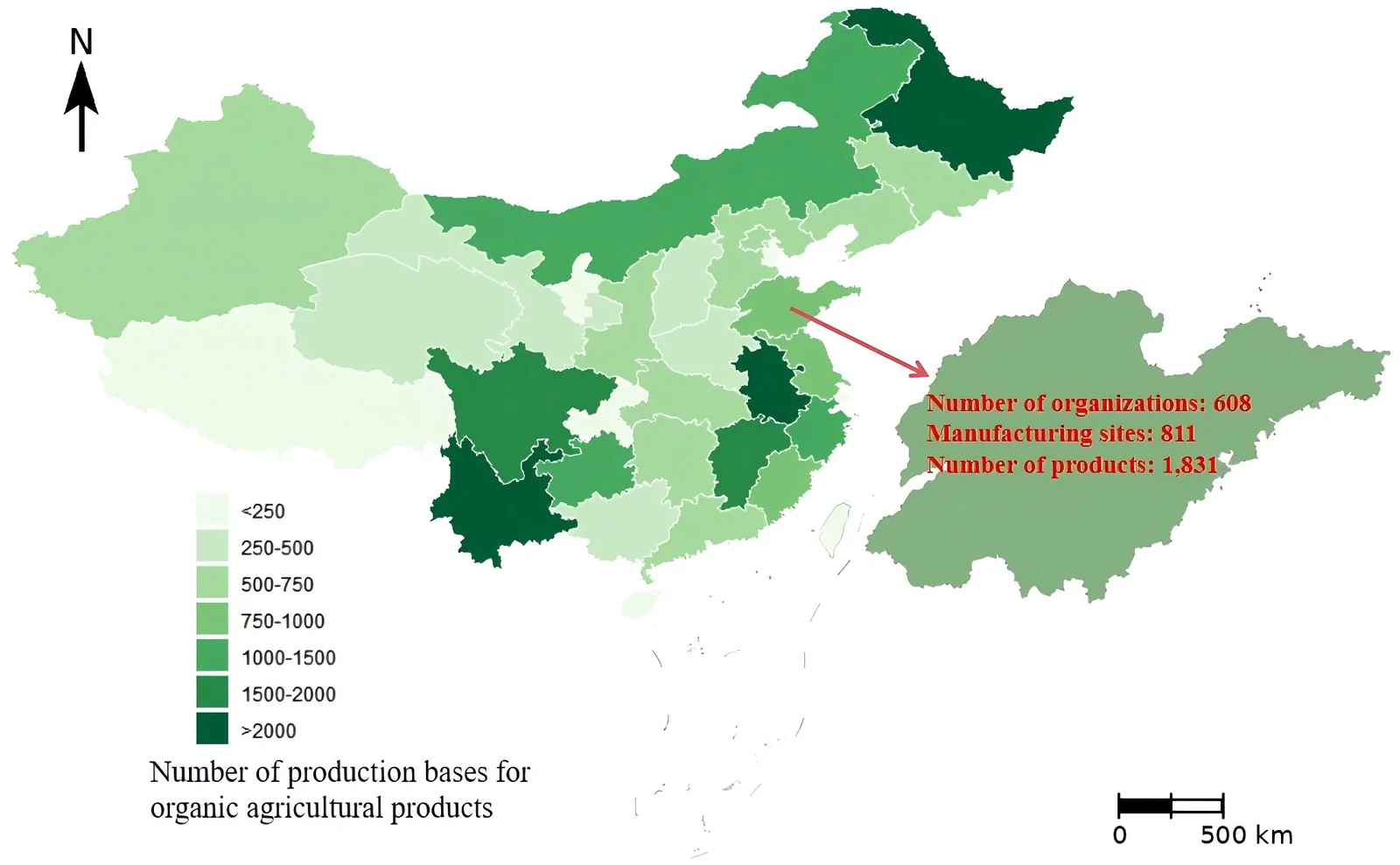
Provincial spatial distribution of certified organic agricultural enterprise production bases in China, 2024. Source: Maps created by the authors using R, based on administrative boundary data from the Global Administrative Areas (GADM) database (https://gadm.org/). Publication of maps in academic articles under a CC BY license is permitted by the GADM license (https://gadm.org/license.html).

(2) Data on regional influencing factors

Regional socioeconomic, agricultural, infrastructural, and demographic variables for the 16 prefecture-level cities during 2014–2023 were compiled primarily from the Shandong Statistical Yearbook) [34] and supplemented, where necessary, by municipal statistical yearbooks, municipal statistical communiqués, government open-data platforms, and administrative records. Standard statistical indicators, including agricultural inputs, regional economic indicators, household income, transportation infrastructure, demographic characteristics, education, and fiscal expenditure, were obtained from the corresponding sections of the provincial or municipal statistical yearbooks. Geographical-indication data were compiled from administrative records of the China National Intellectual Property Administration and the Shandong Administration for Market Regulation. E-commerce indicators were obtained from regional e-commerce statistical or monitoring reports and municipal commerce authorities. For constructed indicators, including the climate suitability index, the underlying variables and calculation procedures are reported separately. Table 1 provides the definition, measurement unit, and specific source category for each variable to ensure variable-level data traceability.

**Table 1:** Indicator Selection and Description.

Dimension	Indicator	Code	Unit	Operational meaning	Data source
Dependent variable	Certified organic enterprise production bases	Y	Number	Spatial presence and organizational participation of certified enterprise-led organic production	Qiyan Social Science Big Data Platform (CCAD), China Food Safety Research Database → Food and Agricultural Product Certification → Organic Product Certificate Information Table
Natural resource endowment	Per capita arable land area	X1	mu/person	Land resource endowment	Shandong Statistical Yearbook 2024 and municipal statistical yearbooks/statistical communiqués
Natural resource endowment	Climate suitability (proxied by annual sunshine duration)	X2	hours	Proxy for climatic suitability for crop growth	Shandong Statistical Yearbook 2024 and municipal statistical yearbooks / meteorological statistical records
Agricultural development foundation	Primary industry value added as a percentage of GDP	X3	%	Relative importance of the primary industry in the regional economy	Shandong Statistical Yearbook 2024; calculated from primary-industry value added and regional GDP where necessary
Agricultural development foundation	Pesticide application	X4	tons	Agricultural chemical-input intensity	Shandong Statistical Yearbook 2024, Agriculture section; supplemented by municipal statistical yearbooks where necessary
Agricultural development foundation	Fertilizer application	X5	tons	Agricultural chemical-input intensity	Shandong Statistical Yearbook 2024, Agriculture section; supplemented by municipal statistical yearbooks where necessary
Economic development foundation	GDP per capita	X6	RMB 10,000/person	Regional economic development level	Shandong Statistical Yearbook 2024, National Accounts section; municipal statistical yearbooks where necessary
Economic development foundation	Urban residents’ per capita disposable income	X7	RMB/person	Household consumption capacity	Shandong Statistical Yearbook 2024, Household Income and Expenditure section; municipal statistical yearbooks where necessary
Economic development foundation	Road length	X8	km	Transport accessibility and logistics infrastructure	Shandong Statistical Yearbook 2024, Transportation section; municipal statistical yearbooks where necessary
Economic development foundation	E-commerce purchases	X9	RMB 10,000	Digital consumption and market connectivity	Regional e-commerce statistical/monitoring data and municipal commerce authority reports
Social environment	Urbanization rate	X10	Proportion (0–1)	Population concentration and urban development	Shandong Statistical Yearbook 2024 and municipal statistical communiqués
Social environment	Number of Internet users	X11	10,000 persons	Digital connectivity and potential online-market access	Shandong Statistical Yearbook 2024, Posts and Telecommunications section; municipal statistical yearbooks where necessary
Social environment	Number of primary school students	X12	Persons	Demographic and household-structure proxy	Shandong Statistical Yearbook 2024, Education section; municipal statistical yearbooks where necessary
Social environment	E-commerce sales	X13	RMB 10,000	Development of digital sales channels	Regional e-commerce statistical/monitoring data and municipal commerce authority reports
Policy and institutional factors	Number of geographical indication protected products	X14	Number	Place-based quality certification and regional branding	China National Intellectual Property Administration and relevant Shandong provincial/municipal administrative records
Policy and institutional factors	Expenditure on agriculture, forestry and water affairs	X15	RMB 10,000	Public fiscal support for agriculture and rural development	Shandong Statistical Yearbook 2024, Government Finance section; municipal statistical yearbooks and fiscal reports where necessary

(3) Spatial data

The administrative boundary shapefile of prefecture-level cities in Shandong Province was obtained from the Global Administrative Areas database (GADM) and handled using the WGS 84 geographic coordinate reference system (EPSG:4326). After boundary unification and spatial registration, all spatial analyses were conducted in R.

### 2.3 Study region

Shandong Province provides a solid geographical, resource, and institutional foundation for this study.

First, geographical advantages. Shandong is located on the eastern coast of China, at the lower reaches of the Yellow River, and administers 16 prefecture-level cities. It is an important agricultural production base and a major exporter of agricultural products nationwide. The province slopes from southwest to northeast, with diverse landforms including plains, hills, mountains, and coastal zones, forming a wide range of suitable areas for agriculture. With a distinct temperate monsoon climate, an average annual rainfall of 600–950 mm [34], and abundant light and heat resources, Shandong is highly suitable for producing diversified, high-value organic products.

Second, agricultural resource advantages. From an agricultural development perspective, Shandong has a strong agricultural foundation. In 2023, the province’s total agricultural output value reached RMB 870 billion, while its agricultural export value ranked first nationwide. With integrated urban–rural development and the deepening of green ecological concepts, Shandong has formed three major agricultural zones: the Bohai Rim metropolitan agriculture zone, the central Shandong hilly specialty agriculture belt, and the Yellow River floodplain ecological agriculture zone, providing diverse spatial carriers for organic agriculture [35].

Third, institutional environment advantages. Over the past decade, organic agriculture in Shandong has developed rapidly. Between 2014 and 2023, the number of certified organic production bases increased from 395 to 753, while the number of certified products rose from 992 to 1,624. Shandong has been proactive in agricultural greening policies, issuing multiple documents. These policies support brand building, base development, and standardization in organic agriculture.

### 2.4 Spatiotemporal evolution analysis

This study first characterizes the spatiotemporal distribution of enterprise bases from the three-dimensional perspectives of “quantity change, spatial pattern, and dynamic trends,” drawing on Shandong Province's organic agriculture certification data from 2014 to 2023. First, quantitative trend analysis was performed. The number of organic agriculture enterprises and the rate of change were calculated annually in each prefecture-level city to reveal the overall pace of development. Second, spatial distribution visualization was performed. Using R, the number of enterprises was mapped to the 16 administrative units of Shandong Province, creating an intuitive distribution map. Third, dynamic evolution trends were assessed. By combining the trajectory of enterprise quantity changes with the evolution of spatial agglomeration, dynamic trends such as center shift and agglomeration-diffusion were analyzed. This paper can only rely on the number of certified enterprise bases and cannot obtain data on changes in enterprise size, which is one of the limitations of the research. This limitation will be explained at the end of the paper.

### 2.5 Spatial correlation test

Spatial correlation was analyzed using Moran's I. Moran's I is a commonly used indicator for testing the degree of spatial autocorrelation, reflecting the correlation between the attribute value of a geographic unit and the values of neighboring units [30]. It is an important indicator for measuring the spatial correlation of time series data and can be divided into global Moran's I and local Moran's I. This paper mainly uses the global Moran's I to analyze the spatial autocorrelation of enterprise production bases in Shandong Province. The calculation method is: construct an adjacency weight matrix ω, calculate the annual global Moran’s I:


$$\begin{array}{c} I=\frac{n}{\sum_{i=\text{1}}^{n}\sum_{j=\text{1}}^{n}\omega_{ij}}\cdot \frac{\sum_{i=\text{1}}^{n}\sum_{j=\text{1}}^{n}\omega_{ij}\left(x_{i}-\overset{―}{x}\right)\left(x_{j}-\overset{―}{x}\right)}{\sum_{i=\text{1}}^{n}{\left(x_{i}-\overset{―}{x}\right)}^{\text{2}}} \end{array}$$ (1)

Moran’s I (I) ranges from −1–1: when I > 0, it indicates positive spatial correlation (adjacent regions are similar); when I < 0, it indicates negative spatial correlation (adjacent regions are opposite); and when I ≈ 0, it indicates random spatial distribution.

This study constructed a prefecture-level “adjacency matrix” as the basis for spatial weighting, calculated annual Moran’s I values and their Z-test values, and plotted their changing trends to identify whether spatial agglomeration exhibits an “increasing-decreasing” pattern over time.

To further identify the local spatial clustering patterns underlying the global spatial autocorrelation, this study additionally employed Local Moran’s I (LISA) analysis. Unlike Global Moran’s I, which measures overall spatial dependence across the province, Local Moran’s I identifies the specific locations of statistically significant spatial clusters and spatial outliers. Based on the spatial weight matrix of prefecture-level cities, the LISA analysis classifies local spatial associations into four categories: High–High (HH), Low–Low (LL), High–Low (HL), and Low–High (LH). This allows the study to reveal the spatial evolution of core and peripheral areas in enterprise-led organic agriculture development.

### 2.6 Analysis of influencing factors

To identify the key factors influencing the spatial distribution and temporal evolution of certified organic enterprise production bases, this study adopts a complementary two-step strategy. First, Pearson correlation analysis is used as an initial screening tool to describe bivariate associations between the dependent variable and the multi-dimensional indicator system (Table 1). Second, to account for unobserved heterogeneity across cities and common shocks over time, we further estimate TWFE panel regressions, which provide a more rigorous assessment of the robustness of key drivers under controlled conditions.

Pearson’s correlation coefficient reflects the strength and direction of linear association between two variables, serving as descriptive evidence of potential relationships [36]. However, because correlation analysis does not control for confounding factors, the TWFE specification is employed as a complementary approach to validate whether the observed correlations remain stable after controlling for city and year fixed effects.

To move beyond bivariate correlations and further identify the determinants of enterprise-led organic agriculture development, we constructed a balanced city–year panel dataset for 16 prefecture-level cities in Shandong Province covering the period 2014–2023. We then estimated a TWFE model to control for unobserved heterogeneity across cities and common shocks over time [32](Formula 2). The baseline specification is:


$$\begin{array}{c} \text{ln}(Bases_{it}+\text{1})=\alpha +\beta^{\prime }{𝐗}_{it}+\mu_{i}+\lambda_{t}+\varepsilon_{it} \end{array}$$ (2)

Specifically, city fixed effects ($\mu_{i}$) account for time-invariant characteristics such as geographic location, historical development paths, and persistent institutional differences, while year fixed effects ($\lambda_{t}$) capture province-wide temporal shocks (e.g., macroeconomic fluctuations and policy environment changes) affecting all cities simultaneously. Given that the dependent variable—the number of certified organic enterprise production bases—is a count outcome with a right-skewed distribution, we used ($\text{ln}(Bases_{it}+\text{1})$) as the dependent variable to stabilize variance and reduce the influence of extreme values. Where (${𝐗}_{it}$) represents the time-varying explanatory variables derived from the indicator system (Table 1), and ($\varepsilon_{it}$) is the error term. To address potential within-city serial correlation, standard errors were clustered at the city level. In line with the proposed “Pressure – Transformation – Support – Market” framework, explanatory variables were introduced stepwise across model specifications to examine the robustness of key drivers under different sets of controls. This specification exploits within-city variation over time after netting out time-invariant city factors and common time trends.

As an additional robustness check, we estimated a Poisson pseudo-maximum likelihood (PPML) fixed-effects model. Because the dependent variable represents the annual number of certified enterprise production bases, PPML provides a suitable alternative specification for count outcomes and is less sensitive to heteroskedasticity than log-linear models. The PPML specification includes both city and year fixed effects to maintain consistency with the TWFE framework.

### 2.7 Indicator selection

To further explore the driving mechanism of the spatial layout of organic agriculture in Shandong Province, this paper constructs an influencing factor index system with 15 indicators from five dimensions: natural resources, agricultural foundation, economic development, social environment, and policy support (Table 1).

(1) Natural resource endowment. Whether the base can be formed and expanded stably depends first on the basic element of natural endowment. First, per capita arable land area reflects the regional human-land relationship and land pressure. It is an intuitive indicator to measure the possibility of large-scale operation, the degree of land dispersion and the difficulty of land transfer [37]. Second, the climate suitability index. This study uses the number of sunshine hours as a proxy to describe the basic suitability of the crop growth environment [38]. It is closely related to photosynthetic efficiency, yield stability, quality formation, pest and disease risks, etc. These two indicators can explain the spatial distribution differences of the base from the two paths of “whether it can be planted” and “whether it is planted well,” and the data caliber of the two is stable and the temporal and spatial comparability is good.
(2) Agricultural development foundation. First, the proportion of the primary industry reflects the structural weight of agriculture in the regional economic structure. It is related to industrial division of labor, labor allocation, and fiscal attention [39]. Second, although the total amount of pesticide application is not a pure indicator of greenness, it can be used as an observable proxy for the scale of agricultural operations and the intensity of farming [40]. Third, the amount of fertilizer applied, similar to the amount of pesticide applied, can further test the robustness of the impact of pesticide application on the number of bases, and both belong to scale agents [41].
(3) Economic development foundation. The base is not only a production unit, but also a node connecting industrial organization and the market, which is inseparable from payment capacity, logistics accessibility and factor flow. First, GDP per capita, which represents the overall development stage and the potential upper limit of capital conditions. Second, residents’ disposable income, which more directly corresponds to the effective demand and payment capacity of the local market, is particularly critical for high-quality, branded organic agricultural products. Third, road mileage, which measures accessibility, affects the acquisition, distribution and radiation range of production materials [42]. Fourth, e-commerce purchase amount. With the popularization of e-commerce, new consumption patterns have an important impact on the development of organic agriculture [43]. Consumers are increasingly focusing on consumption in e-commerce channels.
(4) Social environment factors. The social environment affects the employment organization, brand communication and stable demand of the base through population structure, digitalization level and public services. First, the urbanization rate reflects the degree of population spatial concentration, public service supply and factor matching, and has a long-term impact on labor availability and consumption structure [44]. Second, the number of Internet users. In recent years, the Internet has become the “new infrastructure” of agricultural transformation, determining the efficiency of production and marketing information matching, the penetration of live e-commerce, brand reach and the feasibility of contract farming [45]. Third, the number of primary school students is a proxy for family structure and parental preferences, and is also related to the local long-term consumption and labor supply potential [46]. Fourth, e-commerce sales. This indicator can reflect the importance that local companies attach to e-commerce channels [47]. This is also an important emerging sales channel for organic agricultural products and will become increasingly important with the popularization of the Internet.
(5) Policy and institutional factors. First, the number of geographical indication protected products binds regionality, quality standards and market premiums, strengthens recognizability and bargaining power, and reflects the importance that companies and governments attach to brands and quality [48]. Second, government agricultural expenditure directly refers to government resource supply and has a direct impact on base creation, promotion of good varieties and good methods, public brand building, demonstration projects, etc [49].

## 3 Results

### 3.1 Temporal evolution characteristics

To identify the temporal evolution characteristics of organic agriculture in Shandong Province, this paper mapped the number of enterprises to prefecture-level city units and compiled the quantity distribution (Table 2) and trend (Fig 2) for 10 years from 2014 to 2023.

**Table 2:** Statistics on the number of organic agricultural production bases at the prefecture and city levels in Shandong Province (2014-2023).

Year	Total	Min	Max	Mean	St.d	Skewness	Kurtosis
2014	395	4	52	24.69	14.64	0.83	−0.38
2015	300	4	43	18.75	13.48	0.83	−0.82
2016	337	2	51	21.06	15.42	0.93	−0.20
2017	428	6	65	26.75	18.51	1.04	0.04
2018	372	4	74	23.25	19.94	1.49	1.66
2019	303	2	77	18.94	18.29	2.38	6.76
2020	233	2	43	14.56	11.47	1.41	1.68
2021	250	1	40	15.63	12.13	1.08	0.04
2022	224	2	45	14.00	11.01	1.64	3.21
2023	753	9	111	47.06	32.95	0.68	−0.99

Source: Authors’ calculations based on certification records obtained from the Qiyan Social Science Big Data Platform (CCAD).

**Fig 2:**
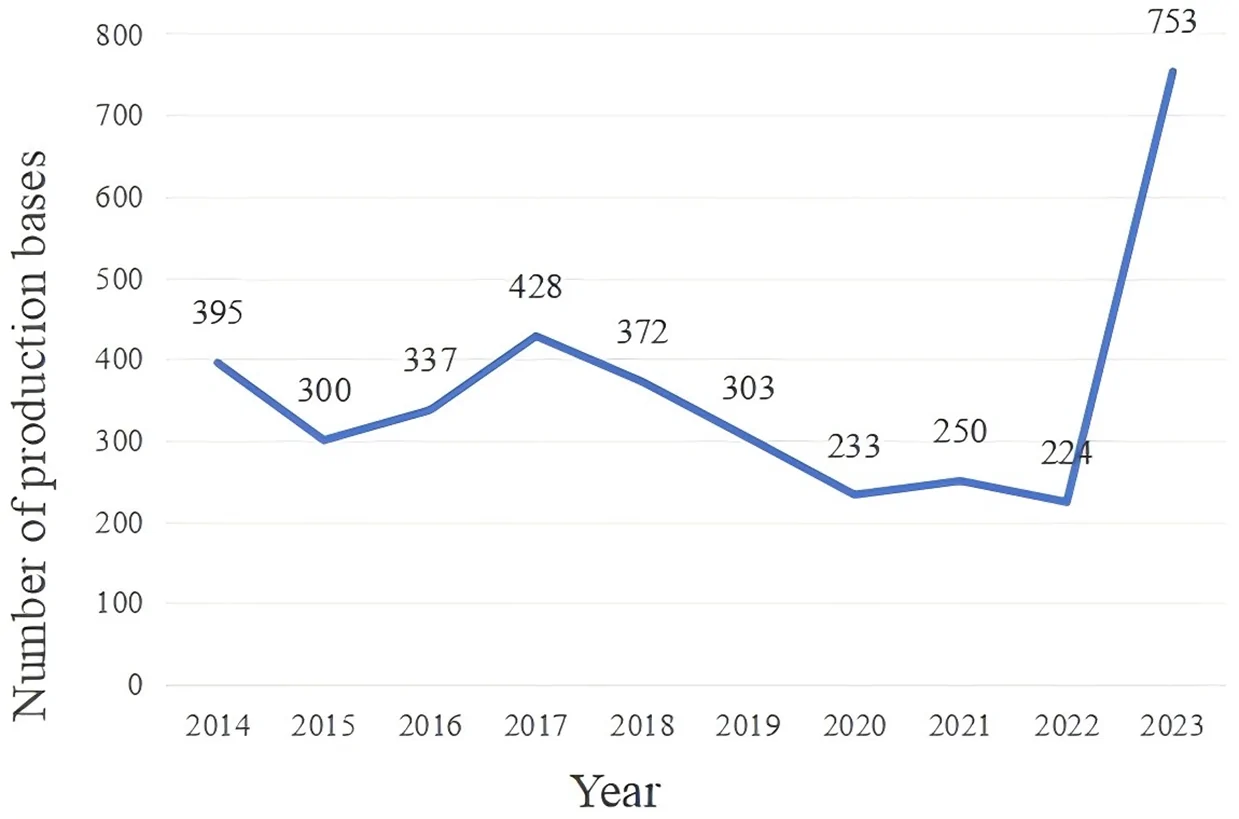
Trends in the number of organic agricultural production bases in Shandong Province (2014–2023).

First, overall scale. The total number of organic agricultural production bases in Shandong Province increased from 395 in 2014–753 in 2023, a net increase of 358 over the past decade, a growth rate of approximately 90.6%. During the same period, the average number of bases per city rose from 24.69 to 47.06 (a 90.6% increase), indicating not only overall expansion but also increasing penetration. The total number of enterprises did not increase linearly, but rather experienced fluctuations. After reaching a peak of 428 in 2017, it began to decline, reaching a low point of around 230 between 2020 and 2022. The most notable feature was the surge in 2023, reaching a total of 753, a 236% increase over the previous year and far exceeding any other year. Over the past decade, the development of organic agricultural enterprises in Shandong Province has consistently exhibited significant spatial imbalance.

Second, the degree of concentration and differentiation. The trend of the average value is roughly consistent with the total number, reaching a peak of 47.06 in 2023, indicating the highest number of production bases per spatial unit. The standard deviation fluctuated upward from 14.64 in 2014 to 32.95 in 2023, indicating widening regional imbalances. The gap between regions with a large number of certified enterprises and those with few or none is becoming increasingly pronounced.

Third, characteristics of the development stage. From 2014 to 2017, there was a period of fluctuating growth. From 2018 to 2022, there entered a period of plateauing adjustment. The decline in the total number may be due to factors such as rising certification standards, market elimination, or the pandemic. A new phase of explosive growth began in 2023.

Fourth, changes in the landscape in 2023. Although the total amount of data in 2023 is huge, its skewness is low (0.68) and kurtosis is negative (−0.99), indicating that although a large number of newly certified enterprises have emerged, these new enterprises may no longer be concentrated in the traditional core areas, but have a tendency to spread to more regions, although the absolute difference between regions (Std = 32.95) is still expanding.

### 3.2 Spatial evolution characteristics

The spatial distribution of certified organic agricultural production bases in Shandong Province exhibits a highly pronounced and increasingly intensified imbalance: a “strong east, weak west” pattern (Table 3 and Fig 3). The Jiaodong Peninsula region (Yantai City, Weihai City and Qingdao), forms a dense core cluster of organic agriculture. Yantai, as the undisputed industry leader, has repeatedly topped the provincial list for the number of production bases, reaching 111 in 2023, becoming the only prefecture-level city in the province with over 100, demonstrating its deep industrial foundation and sustained growth momentum. Weifang, playing a stabilizing role, maintains consistently high figures, forming a “dual engine” of industrial development alongside Yantai. Qingdao, a key coastal economic center, achieved significant growth in 2023, with the number of bases soaring to 96, further consolidating and strengthening the Jiaodong region's dominant position.

**Table 3:** Spatial distribution of organic agricultural production bases in various cities in Shandong Province (2014–2023).

City	2014	2015	2016	2017	2018	2019	2020	2021	2022	2023
Jinan	17	20	22	13	9	5	6	8	13	27
Qingdao	39	35	33	38	32	25	10	30	25	96
Zibo	20	7	13	10	8	7	2	4	4	22
Zaozhuang	4	4	2	6	6	6	2	1	3	9
Dongying	14	8	12	18	13	15	10	11	2	11
Yantai	50	43	51	65	56	77	43	39	45	111
Weifang	52	40	44	61	74	40	36	40	27	88
Jining	23	7	10	21	27	20	6	13	6	24
Tai'an	21	15	16	33	20	9	14	12	9	43
Weihai	15	11	16	19	17	17	12	13	9	75
Rizhao	10	11	24	16	21	12	18	10	13	70
Linyi	47	40	49	52	47	24	23	29	21	73
Dezhou	27	23	15	28	9	14	12	15	10	30
Liaocheng	19	12	5	8	4	7	21	13	12	26
Binzhou	24	18	19	26	19	23	9	6	15	27
Heze	13	6	6	14	10	2	9	6	10	21

**Fig 3:**
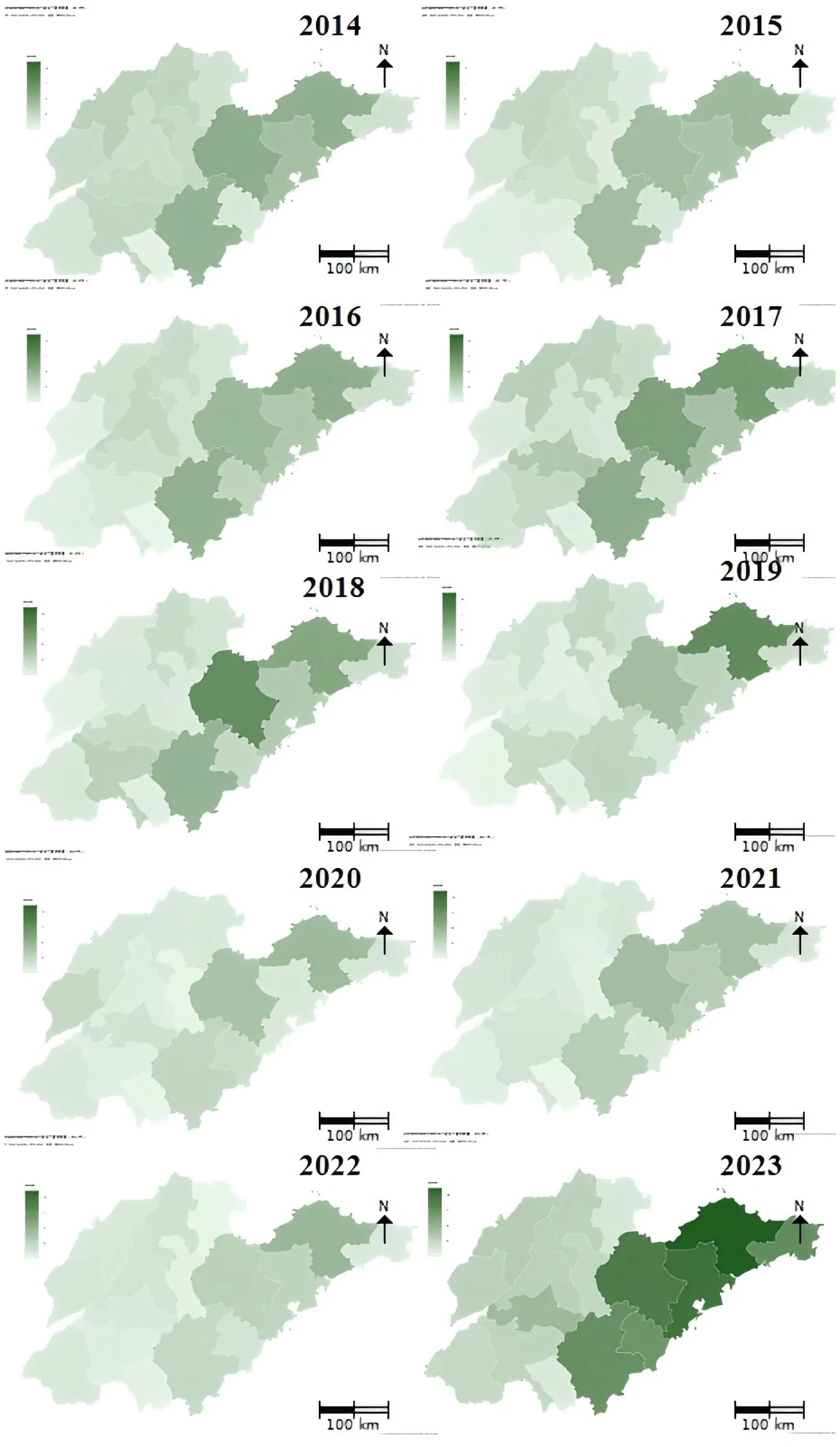
Spatiotemporal evolution of organic agricultural production bases in Shandong Province (2014–2023). Source: Maps created by the authors using R, based on administrative boundary data from the Global Administrative Areas (GADM) database (https://gadm.org/). Publication of maps in academic articles under a CC BY license is permitted by the GADM license (https://gadm.org/license.html).

### 3.3 Global spatial correlation

The global Moran’s I from 2014 to 2023 (Table 4) reveal the distribution of organic agricultural bases in Shandong Province exhibits spatial agglomeration characteristics. First, the spatial distribution has gradually evolved from a moderately concentrated pattern to a highly concentrated one. Between 2014 and 2023, the spatial distribution exhibited significant positive spatial autocorrelation, and the agglomeration effect continued to strengthen. The global Moran's index steadily increased from 0.215 (p < 0.05) in 2014 to 0.344 (p < 0.001) in 2023, a 60% increase over the past decade. The index for all years passed the significance test (p values below 0.05). Second, the spatial pattern exhibits a stable core–periphery structure. Yantai, Qingdao, and Weifang in the Jiaodong Peninsula consistently form the core concentration area, and Weihai and Rizhao have risen in the later period, strengthening the coastal organic development belt. In contrast, western and southern cities such as Heze, Liaocheng, and Zaozhuang remain peripheral areas with persistently low base counts. The number of production bases in these areas have long remained at relatively low levels and the gap with the core region continues to widen. Third, the spatial pattern also exhibits a path dependence effect. Base the data from 2022 to 2023, we find that 321 out of 529 newly certified bases (60.7%) were concentrated in the five Jiaodong cities (Qingdao, Yantai, Weifang, Weihai, and Rizhao), indicating strong path dependence.

**Table 4:** Global spatial autocorrelation statistics of organic agricultural production bases in Shandong Province (2014-2023).

Year	Moran's I	Expected value	Variance	Z-score	P-value	Significance
2014	0.215	−0.0667	0.023	2.35	0.019	*
2015	0.198	−0.0667	0.024	2.12	0.034	*
2016	0.224	−0.0667	0.023	2.46	0.014	*
2017	0.245	−0.0667	0.022	2.68	0.007	**
2018	0.267	−0.0667	0.021	2.91	0.004	**
2019	0.312	−0.0667	0.020	3.41	0.001	***
2020	0.287	−0.0667	0.021	3.12	0.002	**
2021	0.276	−0.0667	0.022	2.99	0.003	**
2022	0.301	−0.0667	0.020	3.28	0.001	***
2023	0.344	−0.0667	0.019	4.18	<0.001	***

Source: Calculated and visualized by the authors using R based on GADM administrative boundary data.

To further explore the local structure of spatial clustering, Local Moran’s I (LISA) analysis was conducted for representative years (2014, 2019, and 2023) (Table 5). The results reveal a gradual consolidation of High–High (HH) clusters in the Jiaodong coastal region (Fig 4). In the early stage (2014), significant local clustering was relatively weak, and Rizhao mainly appeared as a Low–High (LH) outlier. By 2019, Qingdao emerged as a significant High–High cluster, indicating the initial formation of a coastal core area. In 2023, Qingdao, Yantai, and Rizhao formed stable High–High clusters, while several western and southern cities remained Low–Low or peripheral areas. These findings indicate that the strengthening global spatial autocorrelation was primarily driven by the increasing concentration of enterprise-led organic agriculture in the Jiaodong Peninsula.

**Table 5:** Significant Local Moran’s I (LISA) clusters in representative years.

Year	City	Cluster type	Local Moran I	p-value
2014	Rizhao	Low-High	−1.559	0.005
2019	Qingdao	High-High	0.465	0.015
2023	Qingdao	High-High	2.049	0.013
2023	Yantai	High-High	2.467	0.013
2023	Rizhao	High-High	0.870	0.013

Notes: Only statistically significant local clusters are reported. LISA results are based on 999 random permutations.

**Fig 4:**
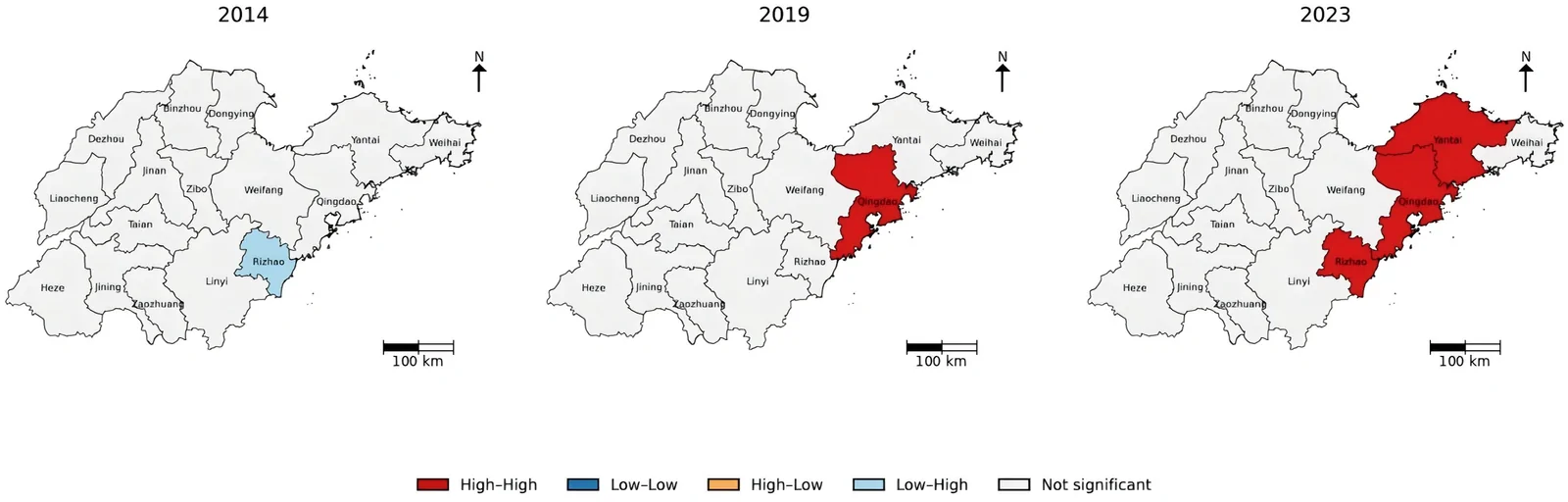
Local Moran’s I (LISA) cluster maps of certified organic enterprise production bases. Source: Maps created by the authors using R, based on administrative boundary data from the Global Administrative Areas (GADM) database (https://gadm.org/). Publication of maps in academic articles under a CC BY license is permitted by the GADM license (https://gadm.org/license.html).

### 3.4 Identification of influencing factors

To provide preliminary evidence on how the proposed determinants are associated with the spatial evolution of certified organic enterprise production bases, we first report Pearson correlation results between the dependent variable and the indicators in Table 6. However, correlation estimates do not control for unobserved heterogeneity across cities or common shocks over time. Therefore, we further employ TWFE panel regressions as a complementary and more rigorous test of the proposed “Pressure – Transformation – Support – Market” mechanism (Table 7).

**Table 6:** Correlation coefficients between the number of production bases of organic agricultural enterprises in Shandong Province and influencing factors.

Indicators	Pearson r	95% CI Lower Limit	95% CI Upper Limit	Spearman ρ	FDR_q (Pearson)	FDR_q (Spearman)	Significance (Pearson)	Significance (Spearman)
X4	0.4741	0.3443	0.5862	0.4566	0.0000	0.0000	***	***
X14	0.4270	0.2912	0.5460	0.2270	0.0000	0.0065	***	***
X15	0.4079	0.2698	0.5295	0.4090	0.0000	0.0000	***	***
X8	0.3522	0.2084	0.4811	0.3437	0.0000	0.0000	***	***
X11	0.3476	0.2034	0.4771	0.3156	0.0000	0.0001	***	***
X9	0.3249	0.1788	0.4571	0.3636	0.0001	0.0000	***	***
X13	0.3007	0.1527	0.4356	0.3384	0.0002	0.0000	***	***
X2	0.2918	0.1431	0.4276	0.3360	0.0003	0.0000	***	***
X7	0.2639	0.1134	0.4026	0.1879	0.0012	0.0260	***	**
X5	0.2631	0.1126	0.4019	0.2972	0.0012	0.0003	***	**
X12	0.1539	−0.0013	0.3018	0.1549	0.0710	0.0669	*	*
X6	0.1445	−0.0109	0.2931	0.1529	0.0853	0.0669	*	*
X10	0.1383	−0.0173	0.2873	0.0836	0.0937	0.3142	*	*
X1	−0.1012	−0.2524	0.0548	0.0175	0.2172	0.8258		
X3	−0.0608	−0.2139	0.0953	−0.1101	0.4450	0.1912		

Note: Significance levels are based on FDR-adjusted q-values: * q < 0.10, ** q < 0.05, *** q < 0.01.

**Table 7:** TWFE regression results.

Variable	Model (1)	Model (2)	Model (3)
X4 (Pesticide use)	0.000000 (0.000032)	0.000059** (0.000028)	0.000021 (0.000042)
X14 (GI products)	0.0570 (0.0421)	0.0420 (0.0334)	0.0587** (0.0280)
X15 (Agri exp.)	−0.000001*** (0.000000)	−0.000002*** (0.000000)	−0.000002*** (0.000000)
X8 (Road length)		0.000123*** (0.000032)	0.000149*** (0.000038)
X11 (Internet users)		0.0013 (0.0011)	−0.0005 (0.0013)
X7 (Income)			−0.000007 (0.000023)
X9 (E-commerce purchases)			0.00000004** (0.00000002)

**Notes:** Dependent variable: ln(Bases + 1). Two-way fixed effects: City and Year included in all models. Cluster-robust standard errors at the city level in parentheses. Significance levels: *** p < 0.01, ** p < 0.05, * p < 0.10. Observations: 160 (16 cities × 10 years). Coefficients can be interpreted as semi-elasticities with respect to ln(Bases+1). Some coefficients appear numerically small due to variable scaling/units in the original statistical yearbook data.

The TWFE results consistently highlight the importance of support and market-related conditions. Fiscal expenditure on agriculture, forestry and water affairs (X15) remains statistically significant across all specifications; however, its estimated coefficient is negative in the TWFE models, which may reflect compensatory fiscal allocation toward lagging areas or potential reverse causality or endogeneity between organic development and policy support. Transport accessibility (road length, X8) is also strongly and significantly positive once included, suggesting that infrastructure foundations are essential for scaling enterprise-led organic production bases. In the full model, the number of GI-protected agricultural products (X14) becomes significantly positive, and e-commerce purchases (X9) also show a positive and significant association, implying that place-based institutional advantages and market connectivity jointly contribute to organic base expansion. By contrast, disposable income (X7) and internet users (X11) are not statistically significant in the full specification, suggesting that their independent contributions may be absorbed by other correlated factors when controls are jointly considered.

Because the dependent variable is a count outcome, we additionally estimated a PPML fixed-effects model as a robustness check (Table 8). The PPML estimates remain broadly consistent with the TWFE results for the most robust explanatory variables, particularly fiscal expenditure, road infrastructure, and e-commerce connectivity. The coefficient of GI-protected products remains positive but loses statistical significance under the PPML specification, suggesting that the branding effect should be interpreted more cautiously. Overall, the robustness checks indicate that the main conclusions are not solely driven by the log-linear specification adopted in the baseline TWFE models.

**Table 8:** Robustness checks.

Variable	Baseline TWFE	Drop-2023 TWFE	PPML FE
X15 Fiscal expenditure	−1.995e-06*** (3.911e-07)	−1.958e-06*** (4.359e-07)	−1.565e-06*** (3.476e-07)
X8 Road infrastructure	0.0001*** (3.774e-05)	0.0002*** (4.668e-05)	0.0001*** (2.809e-05)
X9 E-commerce purchases	2.177e-08** (9.122e-09)	2.018e-08** (8.668e-09)	1.932e-08** (9.466e-09)
X14 GI-protected products	0.0587** (0.0280)	0.0538** (0.0270)	0.0567 (0.0396)
X7 Income	−6.584e-06 (2.288e-05)	−8.944e-07(2.033e-05)	6.324e-06 (2.204e-05)
X11 Internet users	−0.0005 (0.0013)	−0.0004 (0.0013)	−0.0011 (0.0014)

Notes: Baseline TWFE corresponds to the final model in Table 7. Drop-2023 excludes 2023. PPML FE is estimated for count outcomes. City and year fixed effects are included in all models. Cluster-robust standard errors at the city level are in parentheses. *** p < 0.01, ** p < 0.05, * p < 0.10.

## 4 Discussion

### 4.1 Space dependence

From a dynamic evolutionary perspective, the spatial development of enterprise-led organic agriculture in Shandong Province exhibits a clear process of cumulative agglomeration and increasing regional polarization. Although the total number of certified production bases expanded substantially over the study period, this growth was not evenly distributed across the province. Instead, development increasingly concentrated in the Jiaodong coastal region, particularly in cities such as Yantai, Qingdao, Weihai, and Rizhao, while western and southern inland areas remained relatively peripheral. This pattern indicates that enterprise-led organic agriculture does not diffuse uniformly across space, but rather tends to accumulate in regions with stronger pre-existing advantages, including infrastructure accessibility, market connectivity, institutional support, and industrial foundations. The explosive expansion observed in 2023 further reinforced this tendency, suggesting that newly emerging development opportunities were primarily absorbed by already advantaged regions, thereby intensifying the province’s core–periphery structure.

This spatial distribution pattern is consistent with significant positive spatial autocorrelation. Core areas in the Jiaodong Peninsula are geographically contiguous, while several relatively low-intensity areas are also spatially adjacent. The Global and Local Moran’s I results therefore indicate that certified organic production bases are spatially clustered rather than randomly distributed. Such clustering may be associated with shared regional conditions, including agricultural foundations, market accessibility, infrastructure, and institutional environments. However, the present analysis does not directly identify spatial spillover effects between neighboring cities.

In stark contrast to the prosperity of the eastern coastal areas, development in the western and southern inland regions has been relatively slow, creating a distinct “peripheral zone.” Annual figures for cities like Zaozhuang, Heze, and Liaocheng have long hovered at low levels, significantly lagging behind the core regions. It's noteworthy that the scale of organic agriculture development in Jinan, the provincial capital, and Jining, a major economic city, doesn't fully match its economic status. This phenomenon suggests that the development of organic agriculture isn't driven solely by economic aggregate, but rather relies more deeply on local resource endowments, industrial policy guidance, and historical path dependence.

This spatial pattern is the result of the combined effects of natural endowments, economic foundations, industrial policies, and geographical location. The Jiaodong region, with its superior natural conditions, well-developed industrial chain, convenient foreign trade channels, and first-mover policy advantages, has fostered strong spatial spillover effects and agglomeration economies. Meanwhile, western regions, constrained by weak industrial foundations and distant proximity to core markets, struggle to break out of the trap of low-level development. Future policymakers must prioritize this spatial heterogeneity and adopt differentiated regional strategies. On the one hand, they should continue to support the Jiaodong region in developing a world-class organic agricultural cluster. On the other hand, they should promote coordinated regional development and address spatial imbalances through measures such as the “one city, one product” initiative and the East-West collaboration mechanism.

In summary, organic agriculture in Shandong Province exhibits a highly polarized and clustered distribution, with pronounced path dependence. Coordinating balanced regional development and guiding the flow of industrial factors to low-lying areas will be a key policy issue in the future.

### 4.2 Driving mechanism

The combined evidence from Pearson correlations, TWFE regressions, PPML robustness checks, and spatial clustering analysis is consistent with a layered “Pressure–Transformation–Support–Market” interpretive framework for understanding the spatiotemporal evolution of enterprise-led organic agriculture in Shandong Province. Within this framework, production and environmental pressures provide a contextual impetus for green transformation [50], while institutional support, infrastructure accessibility, and market connectivity represent enabling conditions associated with the expansion of certified enterprise production bases. The observed core–periphery structure is also consistent with the cumulative concentration of these regional advantages in the Jiaodong coastal region. These relationships should be interpreted as empirical associations and mechanism-consistent evidence rather than as identified causal pathways.

First, “pressure” represents the contextual impetus for green transition in regions with intensive conventional agriculture and heightened environmental and food-safety concerns. Consistent with this logic, pesticide use intensity is positively associated with base expansion in bivariate relationships, reflecting the background condition of high-input agricultural production systems [51]. However, its effect becomes statistically unstable once policy, infrastructure, and market factors are jointly controlled in the TWFE model. This indicates that pressure alone does not automatically translate into enterprise-led organic growth; rather, it provides motivation for transformation that must be activated through institutional support and practical enabling conditions.

Second, “support” emerges as the most robust pillar for enterprise-led organic development. The TWFE results show that fiscal expenditure on agriculture, forestry and water affairs (X15) remains consistently significant across specifications, indicating a strong linkage between public resource allocation and certified base dynamics. Notably, the coefficient is negative, which may imply that fiscal spending is partly directed toward structurally weaker areas as compensatory support, or that expenditure responds to regional gaps rather than purely driving expansion. Fiscal expenditure on agriculture, forestry and water affairs remains statistically significant across the TWFE and robustness specifications, but its coefficient is consistently negative. This result should not be interpreted as evidence that fiscal support inhibits organic development. One plausible explanation is compensatory allocation, whereby greater public expenditure is directed toward structurally weaker or lagging agricultural areas. Reverse adjustment, in which fiscal resources respond to existing regional development gaps, is another possibility. Because the present observational design does not identify the direction of causality or the specific channels through which fiscal expenditure operates, these explanations remain interpretive rather than empirically verified mechanisms.

Third, “market” and “supporting conditions” jointly shape the spatial concentration of organic bases. Transport accessibility, captured by road length, exhibits a stable and strong positive effect in the TWFE model, underscoring that logistics connectivity and reduced transaction costs are essential for organizing input–output circulation and operating spatially clustered base networks. Moreover, e-commerce purchases are positively and significantly associated with base expansion, whereas disposable income is not significant after accounting for fixed effects. This pattern suggests that market connectivity through digital trade channels may matter more than local purchasing power in explaining where certified bases grow, as enterprises can leverage broader demand beyond the local market through integrated supply chains and online platforms [52]. Meanwhile, the independent contribution of internet users becomes insignificant once transport and e-commerce indicators are included, implying that general digital penetration may overlap with more direct measures of market access. The LISA results further suggest that the Jiaodong Peninsula has evolved from isolated local advantages toward a stable High–High agglomeration zone. This implies that enterprise-led organic agriculture development is increasingly shaped by cumulative regional advantages, including infrastructure connectivity, market accessibility, branding spillovers, and policy concentration effects.

Overall, the combined evidence depicts a layered driving process: external “pressure” creates incentives for transformation, but the observable spatial agglomeration and core–periphery differentiation of enterprise-led organic bases are primarily reinforced through (i) sustained fiscal support and place-based institutional advantages (GI resources), (ii) infrastructure-enabled accessibility, and (iii) market connectivity via e-commerce channels. This integrated mechanism helps explain why the Jiaodong Peninsula core can continuously accumulate advantages and attract organic base expansion over time, thereby intensifying spatial agglomeration and regional differentiation across Shandong.

### 4.3 Limitations

This study still has certain limitations and requires further improvement in future research. First, the certification data used in this paper only records the location and basic information of enterprise production bases, lacking statistics on the actual area of organic farmland, thus failing to directly assess development differences at the scale level of agriculture. Future research could strengthen causal identification by exploiting exogenous policy shocks through difference-in-differences designs, or by employing instrumental-variable strategies and richer enterprise-level microdata to better track firm entry/exit dynamics and investment behavior. Second, this paper mainly uses structural indicators and spatial models to identify influencing factors, while soft factors such as market trust and consumer preferences have not been included in the analysis, which may lead to an incomplete explanation of the spatial evolution mechanism of the industry. Third, certification records are updated annually, and due to the certification cycle, the data may have a time lag, making it difficult to accurately capture the real-time dynamics of enterprises entering and leaving the market. Future research can achieve a more comprehensive understanding of the spatial pattern and organizational evolution of organic agriculture by introducing remote sensing farmland data, enterprise operating scale indicators, and market behavior variables. In addition, although the two-way fixed-effects specification helps control for unobserved city heterogeneity and common time shocks, it remains observational and cannot fully establish strong causal relationships, especially in the presence of potential time-varying omitted variables.

## 5 Conclusions

This study systematically analyzes the spatiotemporal evolution and influencing factors of organic agricultural production bases in Shandong Province from 2014 to 2023, reaching the following key conclusions:

First, from a temporal perspective, Shandong's organic agricultural production bases exhibit distinct phased characteristics over time, with their overall scale experiencing significant growth amidst fluctuations, particularly explosive expansion in 2023.

Second, in terms of spatial pattern, the distribution of bases exhibits significant and continuously increasing agglomeration. A stable “core-periphery” structure has formed spatially. The Jiaodong region forms a persistent core area, while western and southern Shandong remain peripheral areas with comparatively low base intensity.

Third, the empirical results indicate that regional conditions are associated with the spatial differentiation of certified enterprise-led organic production bases in different ways. Road infrastructure and e-commerce purchases show positive and comparatively robust associations with base expansion across the TWFE and robustness specifications, highlighting the relevance of physical accessibility and market connectivity. Fiscal expenditure on agriculture, forestry and water affairs shows a consistent negative association, which may reflect compensatory allocation or reverse adjustment and therefore requires cautious interpretation. GI-protected products are positively associated with base development in the full TWFE model, but this relationship is not statistically significant in the PPML robustness specification. Meanwhile, several conventional resource and socioeconomic indicators do not remain significant after city and year fixed effects are introduced. Taken together, these findings suggest that the evolution of certified enterprise production bases cannot be explained by agricultural resource endowment alone and is also associated with infrastructure, institutional, and market conditions.

Notably, traditional agricultural resource endowment indicators, such as per capita arable land and the proportion of primary industry, have no significant impact, indicating that organic agriculture is no longer simply a resource-intensive industry, but rather a “transformation and upgrading” industry driven by technology, policy, and the market.

This research carries important significance. Practically, it identifies the spatial differentiation patterns of organic production bases in Shandong with precision and unpacks the regionalized mechanisms of multidimensional influencing factors. The findings can directly serve governments at various levels, offering scientific evidence for formulating more refined, region-specific, and targeted development plans and support policies, thereby enhancing the overall quality, efficiency, and equity of organic agriculture. Theoretically, this study enriches the connotations of agricultural geography and regional sustainable development theories within the specific field of organic agriculture, and provides empirical support from a representative agricultural province for constructing a more explanatory theoretical framework for regional organic agricultural development.

## 6 Recommendations

The findings can directly serve governments at various levels, offering scientific evidence for formulating more refined, region-specific, and targeted development plans and support policies. First, we suggest that organic agricultural development should not adopt a “one-size-fits-all” approach but should fully consider regional heterogeneity. For core areas such as the Jiaodong Peninsula, the focus should be on upgrading industrial clusters and extending the value chain. For peripheral areas such as western and southern Shandong, breakthroughs in development bottlenecks are needed through the cultivation of distinctive industries and improved infrastructure. Second, policymakers should focus on the “pressure-transformation” mechanism, transforming environmental constraints into development drivers and achieving a win-win situation for environmental protection and economic development through mechanisms such as ecological compensation. Finally, we should pay attention to the balanced layout of digital infrastructure, and use e-commerce platforms to help backward areas break through geographical limitations and share the dividends of organic agricultural development.

Theoretically, this study enriches the connotations of agricultural geography and regional sustainable development theories within the specific field of organic agriculture by explicitly integrating enterprise-led organizational dynamics into the analysis of spatial evolution. It demonstrates how environmental regulation, market accessibility, and agglomeration externalities jointly shape the spatial configuration of organic production bases, thereby extending existing theoretical discussions beyond land-based or farm-centered perspectives.

## Supporting information

S1 Table Supplementary dataset for the spatiotemporal analysis of organic agricultural production bases in Shandong Province, China (2014–2023). (XLSX)
